# Comparison of nasopharyngeal and guttural pouch specimens to determine the optimal sampling site to detect *Streptococcus equi* subsp *equi* carriers by DNA amplification

**DOI:** 10.1186/s12917-017-0989-4

**Published:** 2017-03-23

**Authors:** Ashley G. Boyle, Darko Stefanovski, Shelley C. Rankin

**Affiliations:** 1 Department of Clinical Studies New Bolton Center, School of Veterinary Medicine, University of Pennsylvania, 382 West Street Rd, Kennett Square, PA 19348 USA; 2 Department of Pathobiology, School of Veterinary Medicine, University of Pennsylvania, 3900 Spruce Street, Room 4036, Philadelphia, PA 19104 USA

**Keywords:** Loop amplification, PCR, Flocked swab, Strangles, *Streptococcus equi*, Horse

## Abstract

**Background:**

*Streptococcus equi* subsp *equi* (*S. equi*) is the cause of “equine strangles” which is a highly infectious upper respiratory disease. Detection of *S. equi* is influenced by site of specimen collection, method of sampling, and type of diagnostic test that is performed. We hypothesized i) that a loop-mediated isothermal amplification (LAMP) assay that targets the *S. equi*-specific *eqbE* gene would be more sensitive than a realtime PCR assay that targets the *S. equi*-specific *seeI* gene and ii) that LAMP of specimens obtained by guttural pouch lavage (GPL) would be more sensitive than LAMP of nasopharyngeal specimens to identify *S. equi* carriers.

**Methods:**

A nasopharyngeal flocked swab, nasopharyngeal wash, and GPL specimen was collected from 44 convalescent horses and the *eqbE* LAMP assay was performed. The *seeI* realtime PCR assay and aerobic culture were also performed on the GPL specimen. Logistic regression was performed to compare sampling sites and test methods (*P*-values ≤0.05 were considered significant).

**Results:**

One of 41 nasopharyngeal flocked swabs, 6/38 nasopharyngeal wash and 24/44 GPL specimens were positive by *eqbE* LAMP. 18/44 GPL specimens were positive by *seeI* PCR and *S. equi* was isolated from 4/44 of these specimens. Detection of *S. equi* DNA was 51 times more likely from the GPL samples than nasopharyngeal samples (OR 51.0, *P <* 0.0001). When *eqb*E LAMP GPL samples were positive, it was eight times more likely that the guttural pouch had any abnormality on endoscopy (OR 8.2, P ≤ 0.005), almost 20 times more likely that mild empyema was found (OR 19.7, *P* ≤ 0.002), and eight times more likely that the *SeeI* PCR was positive for *S. equi* DNA (OR 8.1, *P ≤* 0.006).

**Conclusion:**

This study demonstrates that guttural pouch lavage specimens should be used to detect *S. equi* and that the *eqbE* LAMP assay was comparable to the *seeI* PCR.

**Electronic supplementary material:**

The online version of this article (doi:10.1186/s12917-017-0989-4) contains supplementary material, which is available to authorized users.

## Background

Strangles, caused by *Streptococcus equi* subsp *equi* (*S. equi*)*,* is a highly infectious upper respiratory disease that has a high morbidity rate and poses a high financial burden for the equine industry [1–3]. Up to 20% of recovered horses post-outbreak are persistent carriers of the organism in their guttural pouches [2, 4, 5]. Carrier animals serve as a reservoir for continued spread in the equine population. Bacterial culture and PCR of nasopharyngeal wash and guttural pouch lavage (GPL) specimens have been used to detect *S. equi* for diagnostic testing of clinical suspects and for the detection of carrier animals [2, 3, 5]. Bacterial culture has been documented to have low sensitivity when there are low numbers of *S. equi* [6]. PCR is highly sensitive and specific when the target DNA is present in the specimen. However, during the carrier state organisms may present at a very low number, may be shed intermittently or may be dead; yet their DNA will still be detectable by PCR. Practitioners must obtain multiple sequential samples from convalescing animals in order to ensure a 90% chance of true negatives before comingling with susceptible animals [5].

Detection of *S. equi* carriers is influenced by several factors that include: 1) site of specimen collection (rostral nasal passage, nasopharynx versus guttural pouch; 2) method of sampling (flocked swab, rayon swab, versus wash); 3) culture versus PCR, 4) target gene of the PCR; and 5) DNA amplification method [1, 6–8]. It is well established that *S. equi* is harbored in the guttural pouch and that there is intermittent shedding of organisms into the nasopharynx [3, 5]. The use of flocked swabs during specimen collection has recently been implemented in human medicine because of greater recovery and elution of organisms for testing of both bacterial and viral diseases [9]. Sampling rostral nasal passages of horses with acute strangles infection with flocked nylon swabs did not statistically improve the detection of *S. equi* via PCR and bacterial culture [1]. Sampling the horse nasopharynx with flocked nylon swabs for the detection of *S. equi* has not been investigated.

The identification of a more specific gene target for *S. equi* detection by PCR is constantly under investigation due to the high level of genetic homology of *S. equi* with *Streptococcus equi* subsp. *zooepidemicus* and *Streptococcus pyogenes* [8, 10, 11]*.* In 2008, the *eqbE* gene was shown to be unique to *S. equi* and absent from *S. zooepidemicus*, thus providing a single specific gene target for real time PCR not previously available [11]. Loop-mediated isothermal amplification (LAMP) is a nucleic acid amplification method that is performed at a constant temperature and can detect target DNA in less than 30 min unlike traditional PCR methods that require thermal cycling [12]. LAMP has been used to detect *S. equi* in horses using both the *seM* gene [12] and the *eqbE* gene [13]; the latter assay was shown to be more specific [10].

The reality of strangles disease in the field is that horses are treated with antibiotics to treat the infection and prevent a carrier state as quickly as possible and allow the release of the animal from quarantine. If the *eqbE* LAMP assay is reliable for the detection of *S. equi* using “real world” samples from client-owned animals, then it may be possible to develop a point-of-care diagnostic assay in a hand-held device for stall-side diagnostic testing [14]. We hypothesized that sampling the guttural pouch would be more sensitive than sampling the nasopharynx to identify carriers of *S. equi*. We hypothesized that the *eqbE* LAMP assay would be more sensitive than the real time *seeI* PCR based on the higher specificity of the *eqbE* gene [10] and higher efficiency of LAMP over realtime PCR [15]. The aim of this study was to compare nasopharyngeal and guttural pouch specimens to determine the optimal sampling site to detect *Streptococcus equi* subsp *equi* carriers. Two DNA amplification methods and two gene targets were compared.

## Methods

### Horses

This study was approved by the University of Pennsylvania’s Institutional Animal Care and Use Committee Protocol #805146. All owners had informed consent and agreed to the Widener-New Bolton Center (NBC) Privately - Owned Animal Protocol #1311–1. This study was performed using naturally occurring and recovering cases of *S. equi* infection amongst New Bolton Center field service equine patients. Signalment was recorded. Horses were confirmed as previously infected with *S. equi* (nasopharyngeal wash or guttural pouch lavage *S. equi* positive on PCR or culture, or clinical signs consistent with *S. equi* infection in a confirmed *S. equi* outbreak), and recovered from clinically apparent disease for at least 2 to 3 weeks. Time from the start of clinical signs to sample collection was recorded. Antibiotic history was recorded. All horses were sedated with detomidine (Dormosedan, Zoetis, Florham Park, NJ) 0.015–0.025 mg/kg of body weight for the procedures and were administered one dose of flunixin meglimine (Banamine®, Merck Animal Health, Kenilworth, NJ) 1.1 mg/kg of body weight after the procedure as an analgesic and an anti-inflammatory agent. Samples were collected using 3 different sampling site strategies, and tested using 3 different *S. equi* identification methods.

### Samples

Three samples were collected from each horse in the following order. A nasopharyngeal flocked nylon swab was performed using a 100 mm flocked swab (Floqswab™, Copan Diagnostics, Murietta, CA) taped to a 63.5 cm uterine polyvinyl chloride pipette. The flocked swab was then removed from the pipette and placed in the tube and media of a culturette (BBL™ CultureSwab™, Becton Dickinson, Franklin Lakes, NJ) for transport to the laboratory. Second, a nasopharyngeal wash was performed as previously described by the intranasal administration of 50 ml of sterile saline [16], and third, an endoscopically-guided guttural pouch lavage was performed through a closed system of sterile polyethylene tubing passed through the instrument channel in the sedated, standing horse [17]. The endoscope was cleaned with chlorohexidine (Nolvasan Solution, Zoetis, Persippany, NJ), rinsed with sterile water, and disinfected for 10 min with ortho-phthalaldehyde solution (Cidex OPA, Advanced Sterilization Products, Johnson and Johnson, Switzerland) between each horse on the farm. The following tests were performed on the samples:

1. Nasopharyngeal flocked swab *eqbE* LAMP assay for *S. equi*
2. Nasopharyngeal wash *eqbE* LAMP assay for *S. equi*
3. Guttural pouch lavage (split into 3 aliquots)

- *S. equi* culture
- *S. equi seeI* PCR
- *S. equi eqbE* LAMP assay.

Visual examination of the guttural pouch was performed at the time of sampling through the endoscope on an attached video screen. Briefly, the endoscope was passed via the ventral meatus of the nasal passage, into the nasopharynx, and through the guttural pouch openings sequentially [17].

### Diagnostic testing

All diagnostic testing (*S. equi* culture and *S. equi seeI* PCR) was performed at the University of Pennsylvania NBC Clinical Microbiology Laboratory, which is an AAVLD accredited laboratory.

### *seeI* PCR assay

The *seeI* PCR assay has been previously validated [18]. The limit of detection [6] and its use in detection of *S. equi* in clinical nasopharyngeal wash samples and guttural pouch lavage samples from sick, convalescent, and asymptomatic horses [19] has been published by our laboratory. DNA was extracted from a 1 ml aliquot of the guttural pouch lavage using PrepMan Ultra as described by the manufacturer (Applied Biosystems, Foster City, CA). The lavage fluid was centrifuged (Eppendorf Centrifuge Model 5417C, Germany) for 3 min at 20,000 × *g*, and the pellet was resuspended in 100 μl of PrepMan Ultra and boiled for 10 min at 100 °C. The boiled extract was then diluted 1/100 in nuclease-free water prior to PCR (Fisher Scientific, Pittsburgh, PA) [6].

Realtime PCR was performed with the following primers and probe as previously described by our laboratory [18].

*seeI*-F 5’-CGGATACGGTGATGTTAAAGA -3’

*seeI*-R 5’-TTCCTTCCTCAAAGCCAGA-3’

*seeI* probe 5’-TTTGGCCGCTCCTCTAGATTTCAA-3’

The positive control was *S. equi* ATCC 33398 and positive and negative controls were run with each assay. Each PCR reaction consisted of 20 μl of a commercial l mastermix (Quantifast Pathogen and Internal Control detection kit, Qiagen, Valencia, CA) plus 5 μl of extracted DNA for a final concentration of 3 U *Taq*, 200 μM dNTPs, 4 mM MgCl_2_ and 25 mM HEPES. Amplification began with 8 min at 95 °C, followed by 45 cycles of 20 s at 95 °C and 60 s at 60 °C. No internal amplification control was included.

### Bacterial culture

A culturette (BBL™ CultureSwab™, Becton Dickinson, Franklin Lakes, NJ) was submerged in the vortexed fluid sample, plated on a blood agar plate (Columbia CNA (colistin, nalidixic acid), Becton Dickinson, Franklin Lakes, NJ), aerobically incubated at 35 °C overnight, and read at 24 and 48 h. Beta-hemolytic, organisms that were also catalase negative were subcultured to a blood agar plate and incubated at 35 °C overnight. Isolates were then identified biochemically using a commercial system. Sensititre panel (Trek Diagnostics). In addition to this, isolates were subcultured to Cystine Trypticase Agar (CTA) with lactose and CTA with sorbitol (Becton Dickenson, BBL™ CTA™ Medium) and incubated at 35 °C overnight. *Streptococcus equi* subsp *equi* does not ferment sorbitol or lactose in contrast to *Streptococcus equi* subsp *zooepidemicus*. Presumptive *Streptococcus equi* subsp *equi* isolates were confirmed to be Lancefield group C using a commercial latex agglutination system. (Remel, Lenexa, KS)

### LAMP assay

The *S. equi eqbE* LAMP assay was performed for research purposes at the Matthew J. Ryan Veterinary Hospital (Ryan VHUP) of the University of Pennsylvania Research Microbiology Laboratory [10]. Research personnel at Ryan VHUP were blinded to *seeI* PCR results obtained at NBC and had no information on the clinical status of the animal from which samples were obtained. The *eqbE* LAMP assay was performed using primers that were designed with the PrimerExplorer V4 Software (http://primerexplorer.jp/elamp4.0.0/index.html). The gene sequence used was based on Genbank Accession (http://www.ncbi.nlm.nih.gov/): AM909652, *Streptococcus equi* subsp. *equi* integrative conjugative element ICESE2, strain 4047. The analytical sensitivity of *eqbE* LAMP was determined by using a 10-fold serial dilution (ranging from 5 × 10^2^ ng to 5 × 10^−7^ ng) of ATCC 33398 *S. equi* subsp. *equi* genomic DNA (data shown in Additional file 1: Table S1). The *eqbE* LAMP was carried out in a reaction mixture of 25 μl containing 0.4 μM of each outer primer (F3, B3), 1.6 μM of each inner primer (FIP, BIP), 0.8 μM of loop primer (LoopF, LoopR), 1X Isothermal Master Mix (Pro-Lab Diagnostics, Round Rock, TX), and approximately 20–100 ng of genomic DNA. DNA was extracted from a 1 ml aliquot of the guttural pouch lavage as previously described above (*seeI* PCR assay). Amplification began with 30 min at 65 °C followed by 2 min at 80 °C to terminate the reaction. Positive and negative controls were genomic DNA from ATCC 33398 *Streptococcus equi* subsp. *equi* and reaction mixture without DNA, respectively.

*eqbE* F3 5’- CACATAAAACTACAGTACAAGGT- 3’

*eqbE* B3 5’- GCGAGTATGAGTAATGCCA- 3’

*eqbE* FIP 5’- TAAAGCTTTTTCCCAAGAAGCTTCTGCTGGTGGTCAATTCTCT- 3’

*eqbE* BIP 5’- ATAGGGCTTGGGCTGATGTTAATGCTAAAATAACAACGTGGC- 3’

*eqbE* Loop F 5’- GCGCTTGTCCAACCCGAATA- 3’

*eqbE* Loop B 5’- AAATAGTTGAACGAGTTTGAGCGGT- 3’

The *eqbE* LAMP assay was tested for inclusivity with a panel of 20 well-defined *S. equi* subsp. *equi* isolates and showed 100% sensitivity and specificity. The analytical specificity (exclusivity) was determined using a panel of 14 strains: *S. equi* subsp. *zooepidemicus, S. pneumoniae, S. agalactiae* (2), *Staphylococcus pseudintermedius, Staphylococcus aureus, Enterococcus faecalis, Klebsiella pneumoniae, E. coli, Salmonella typhmimurium, Haemophilus influenzae, Corynebacterium ulcerans* and *Campylobacter fetus* subsp. *venerealis*.

### Guttural pouch endoscopy

All horses had guttural pouch endoscopy performed. The presence or absence of empyema, defined as any gross purulent material in guttural pouch or within the lavaged fluid, in either guttural pouch was recorded. An endoscopic evaluation of “normal” or “abnormal” was recorded for the guttural pouches of each horse. An abnormal guttural pouch was defined when one or more of the following characteristics were found in either the left or the right guttural pouch: empyema, abnormal appearance to the respiratory epithelial lining, and/or visually enlarged retropharyngeal lymph nodes on the floor of the guttural pouch.

### Statistical analysis

All statistical analysis was performed using computerized software (STATA 13.1, College Station, TX). Logistic regression was used to determine the odds ratio (OR) of detection of *S. equi* based on the sampling methods (nasopharyngeal flocked swab, nasopharyngeal wash, and guttural pouch lavage). The status of infection (0, Negative; 1, Positive) was determined using *eqbE* LAMP PCR. A *P*-value of ≤ 0.05 was used to determine the significance of each statistical test. We adjusted for any potential confounding of the use of any antibiotic treatment (systemic or locally in the guttural pouch), the time from the last antibiotic treatment, and the number of days prior to *eqbE* LAMP testing that the sample was taken by determining their statistical significance in the logistic regression model [20].

## Results

**Fig. 1:**
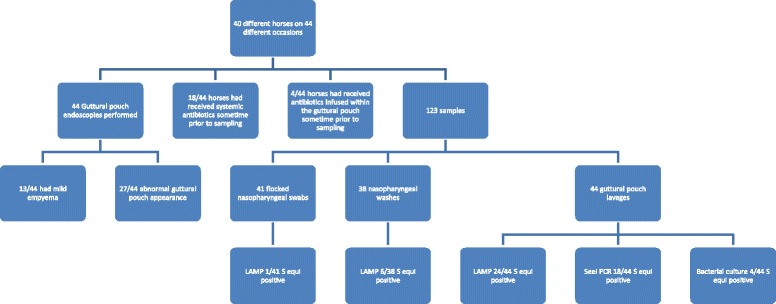
Flow chart of antibiotic history, sample collection, diagnostics, results, and guttural pouchcharacteristics of 40 different *S. equi* convalescent horses obtained on 44 different occassions

One hundred and twenty-three samples were analyzed (Additional file 2: Table S2): 41 nasopharyngeal flocked swabs, 38 nasopharyngeal washes, and 44 guttural pouch lavages were obtained from 40 different horses on 44 separate occasions from all 6 different strangles outbreaks that occurred from November 2013 to November 2014 (Fig. 1). On 3 occasions, the attending clinician neglected to obtain the nasopharyngeal samples and an additional 3 nasopharyngeal wash samples were lost during transport to the research laboratory. Breeds consisted of 20 Standardbreds, 3 Quarter Horses, 9 Thoroughbreds, 2 Warmbloods, 5 draft horses, and one miniature horse. Seventeen horses were female, 16 were geldings, and 7 were male. The median age was 3 years (Interquartile Range [IQR] 1 to 3 years). The median time from start of clinical signs to sampling collection was 3 months (IQR 1 to 3 months).

A total of 31/123 samples (41%) were positive by *eqbE* LAMP: 1/41 nasopharyngeal flocked swab (2%), 6/38 (16%) nasopharyngeal wash and 24/44 (55%) guttural pouch lavages. Eighteen of 44 (41%) guttural pouch lavage samples were positive by *seeI* PCR. The median cycle threshold (C_T_) value was 34.32 (IQR 31.71–36.95). A *seeI* real-time PCR sample was considered negative if the C_T_ value was ≥ 40 cycles. *S. equi* was isolated via bacterial culture from 4/44 (9%) guttural pouch lavage samples [21, 22], all of which were also positive by *seeI* PCR and *eqb*E LAMP. All horses that had a positive sample from the nasopharynx (either flocked swab or wash) were also positive in the guttural pouch. Seventeen (39%) and 27 (61%) of 44 horses had normal and abnormal endoscopic examinations of the guttural pouch, respectively. Thirty-one (70%) of 44 horses had no empyema whereas 13 (30%) of 44 horses had very mild empyema found on endoscopic examination of the guttural pouches. One horse required guttural pouch lavage to clear the particulate matter for clinical reasons. One of 44 horses was positive by *seeI* PCR and had a normal endoscopic examination with no empyema.

Eighteen of 44 horses (41%) had a treatment history of systemic antibiotics: 12 (67%) received procaine penicillin G (Agri-cillin, Zoetis, Florham Park, NJ), 4 (22%) received ceftiofur (Excede, AgriLabs, St Joseph, MO), and 3 (17%) received trimethoprim-sulfamethoxazole (Qualitest, Charlotte, NC). Four of the 44 horses (9%) (4/18 [22%] horses previously treated with systemic antibiotics) had a treatment history of antibiotic usage in the guttural pouch with penicillin-dihydrostreptomycin (Quartermaster, Pharmacia & Upjohn Company, Kalamazoo, MI). In the 18 horses that were treated with antibiotics (systemic or locally within the guttural pouch), all antibiotic treatments finished a median of 3 weeks prior to sampling (IQR 1 week to 2.75 months). All samples were sent to Ryan VHUP and *eqbE* LAMP testing was performed a median of 1 day after collection (IQR 1 day). The use of antibiotics and the number of days following their use that sample collection occurred could affect the positive or negative status of the sample. The number of days after sample collection the *eqbE* LAMP testing was performed may affect the viability of the bacteria within the sample. All three independent variables were added to the 4 logistic models since they may confound the associations between *eqbE* LAMP and the outcomes.

The first logistic model estimated the association between *eqbE* LAMP and the location of sampling. When using the *eqbE* LAMP assay on 123 total samples, the guttural pouch lavage was 51 times more likely to be positive for *S. equi* DNA than the nasopharyngeal flocked swab and the nasopharyngeal wash samples (OR 51, P ≤ 0.0001, 95% CI 6.3–416.6). When the *eqb*E LAMP results of 44 guttural pouch lavage samples were positive, the guttural pouch was eight times more likely to have any abnormality on endoscopy (OR 8.2, P ≤ 0.005, 95% CI 1.9–35.2), almost 20 times more likely to find mild empyema (OR 19.7, P ≤ 0.002, 95% CI 3.0–129.2), and the *SeeI* PCR was eight times more likely to be positive for *S. equi* DNA (OR 8.1, P ≤ 0.006, 95% CI 1.8–36.3). Table 1 shows the sensitivity, specificity, and receiver operator curves for all four logistic models. All models were normalized for the number of days before *eqbE* LAMP testing, antibiotic use, and time from last antibiotic treatment to sample collection.

**Table 1:** Sensitivity and specificity comparing 1) 123 *eqb*E LAMP results to the sample type (guttural pouch lavage (GPL) versus not [nasopharyngeal wash, or nasopharyngeal flocked swab]); 2) *eqbE* LAMP results from 44 GPL samples to abnormal guttural pouch endoscopic findings; 3) *eqbE* LAMP from 44 GPL samples to empyema found on guttural pouch endoscopy; 4) *eqbE* LAMP to *seeI* PCR from 44 GPL samples

		*eqbE* LAMP		Total	Sensitivity	Specificity	PPV^b^	NPV^c^	Correctly Classified	ROC^d^
		Positive	Negative							
GPL^a^ Sampling Method	Yes	24	7	31	77%	78%	55%	91%	78%	0.83
	No	20	72	92						
	Total	44	79	123						
Abnormal Guttural Pouch Endoscopy	Yes	20	7	27	74%	70%	80%	63%	72%	0.80
	No	5	12	17						
	Total	25	19	44						
Guttural Pouch Empyema	Yes	12	1	13	92%	61%	50%	95%	70%	0.85
	No	12	19	31						
	Total	24	20	44						
*seeI* PCR	Positive	15	3	18	83%	65%	63%	85%	73%	0.78
	Negative	9	17	26						
	Total	24	20	44						

The cutoff probability for sample type is ≥ 0.5

^a^Guttural Pouch Lavage

^b^Positive predictive value (based on the detection of *S. equi* DNA and not on the presence of live organisms)

^c^Negative predictive value

^d^Receiver operator curve

All analyses are adjusted for antibiotics usage and the time from last antibiotic treatment by determining if they were significant confounders within the logistic regression via STATA

## Discussion

This study provides strong evidence that the *eqbE* LAMP assay performed with guttural pouch specimens is more sensitive for the detection of *S. equi* than nasopharyngeal flocked swab or a nasopharyngeal wash taken from the same horse at the same time. The 2005 American College of Veterinary Internal Medicine Consensus Statement recommendation for the detection of *S. equi* carriers is to obtain 3 negative nasopharyngeal washes or swab PCR samples over a 3 week period in order for an animal to be considered free of the organism. If the animal is found to be positive, endoscopic examination of the guttural pouch is strongly recommended [5]. The data presented here show that a single guttural pouch lavage nucleic acid amplification test in conjunction with visual examination of the guttural pouches in the convalescent period provides a cost and time efficiency to determine *S. equi* status of an animal

*eqbE* LAMP was found to have acceptable discrimination when *seeI* PCR is considered as a true measure of outcome (ROC 0.78). The sensitivity and specificity of *eqbE* LAMP were lower when compared to the *eqbE* real time PCR results reported by North, 95% and 86%, respectively [10]. This is also true when compared to the triplex quantitative PCR reported by Webb, *et al.* that reported a sensitivity of 94% and specificity of 97% [8]. These previous studies compared samples from horses at all stages of disease (acute, subacute, convalescent) and from all types of samples (abscess aspirates, rostral nasal swabs, nasopharyngeal swabs, nasopharyngeal washes and guttural pouch lavages) which inherently means that the bacterial counts (and therefore gene copies) varied from very high (samples with 10^8^ bacteria in the acute horses and abscess aspirates) to very low (potentially as low as 10 colony forming units in washes of convalescent horses) [6]. This study provides validation for the use of *eqbE* LAMP assay on the typical samples that are tested at the end of an outbreak to clear a patient/farm from quarantine, the guttural pouches of outwardly healthy convalescent horses that have very low bacterial counts but that could still be infective. The GPL culture that was performed adhered to the diagnostic lab protocol and a 10 μl aliquot was plated to the CAN plate. This volume is considerably less than the 1 ml that was centrifuged for DNA extraction and this could have affected the culture results for those samples (23) that were positive on DNA amplification and culture negative. An important incidental finding is that the culture data shows a massive die-off occurred in the guttural pouch in the interval between acute phase strangles and convalescence and is consistent with recent studies in the UK showing *S. equi* undergoes genetic decay in the guttural pouch including loss of genes necessary for virulence and infectivity [23, 24]. This study found flocked swab sampling of the nasopharynx to be a poor method for testing with the *eqbE* LAMP PCR to detect *S. equi* in outwardly healthy convalescent horses. This is in contrast to the improved bacterial recovery that has been found in human medicine sampling and laboratory processing when using flocked swabs [9]. Other veterinary researchers have found that the use of flocked nylon swabs did not improve the detection of *S. equi* by PCR and bacterial culture of the rostral nasal passages in acute strangles infection [1]. This is the first report showing the lack of performance of flocked swabs in the nasopharynx and the convalescent horse.

At the start of this study, the triplex PCR developed by Webb et al. [8] was published, but was not available for research use due to commercial patenting. *seeI* is a gene that encodes a superantigenic toxin [25] that is a virulence factor of *S. equi* [26]*.* Concerns with the *seeI* PCR include a potential lack of specificity between *S. equi* subsp *equi* and subsp *zooepidemicus* due to shared genes for superantigens that may lead to false positive results with some *S. zooepidemicus* strains [27]. In addition, *S. equi* isolated from some persistent carriers have been shown to have deletions in the variable region of the *seM* gene that leads to a false negative test result, although deletions of *eqbE* have also been documented [23, 27]. We attempted to address these limitations by comparing the *eqbE* LAMP results to alternative phenotypic or clinical gold standards (empyema and other abnormalities found on guttural pouch endoscopy) that would make a clinician suspicious that a *S. equi* convalescent horse is still positive. Guttural pouch abnormalities including empyema were a good predictor of a positive *eqbE* LAMP assay result. Positive LAMP or PCR in normal guttural pouches may be the result of biofilm on the respiratory epithelium [28]

Ideally, performing *seeI* PCR on all 123 samples, including all the nasopharyngeal samples would have provided a larger number of samples in which to compare the tests, but was beyond the financial scope of this study. The study met its power calculation recommendations of 33 samples. We did not include the traditional swab as an additional sample collection method, but nasopharyngeal wash has already been shown to be more sensitive than the traditional nasopharyngeal swab [1]. It would have been ideal to compare 3 nasopharyngeal washes over 3 separate weeks in the same horse to one guttural pouch of that horse, but this would be difficult due to the prolonged sampling period and quarantine necessary for this infectious disease in client-owned animals. The current strangles consensus statement is currently under revision to reflect the research advances over the last 11 years. It is known that *S. equi* is intermittently shed from the guttural pouch into the nasopharynx [3]. Horses that have had 3 PCR negative consecutive nasopharyngeal samples have been shown to infect naïve herdmates [28]. Unfortunately, it is not yet possible to quantitatively predict the risk of extended survival of live *S. equi* in the abnormal empyemic guttural pouch. The purpose of this study was to examine the efficacy of *eqbE* LAMP in a “real world” situation during the aggressive sample collection time period (at the end of an outbreak on convalescent animals), which includes all the possible confounders associated with treating client-owned animals such as antibiotic treatments and time constraints on quarantines. The statistical analysis attempted to account for these confounders.

## Conclusions

This study demonstrated the guttural pouch as the preferred anatomic site to sample to detect *S. equi* DNA in outwardly healthy convalescent horses using the *eqbE* LAMP assay. When used in conjunction with visual examination of the guttural pouch it provides evidence to eliminate the need for repeat testing with nasopharyngeal washes or swabs, thus saving time and money. Since the LAMP assay that targets the *eqbE* gene has acceptable agreement with the *seeI* PCR, further field verification of the *eqbE* LAMP in a point-of-care device [14] that will enable stall side testing performed by the field veterinarian is warranted.

## Additional files

Additional file 1: Limit of detection for *eqbE* LAMP assay was determined via a “standard curve” as 0.005 ng of DNA using *S. equi* ATCC 33398. (DOCX 23 kb)

Additional file 2: Raw data of 123 samples (nasopharyngeal flocked swabs, nasopharyngeal washes, and gutturalpouch washes) from 40 different *S. equi* convalesecent horses tested for *S. equi* via *SeeI* PCR and *eqbE* LAMP on 44 different dates. (XLSX 33 kb)

## Abbreviations

C_T_: Cycle threshold
LAMP: Loop mediated isothermal amplification
NBC: New Bolton Center
Subsp: Subspecies
