# Supplementary material for: Comparison of nasopharyngeal and guttural pouch specimens to determine the optimal sampling site to detect Streptococcus equi subsp equi carriers by DNA amplification
Source: BMC Vet Res. 2017 Mar 23;13:75. doi: 10.1186/s12917-017-0989-4 (PMC5364677; doi:10.1186/s12917-017-0989-4)
Supplement: Supplementary file 1 — Limit of detection for eqbE LAMP assay was determined via a “standard curve” as 0.005 ng of DNA using S. equi ATCC 33398. (DOCX 23 kb) [file 12917_2017_989_MOESM1_ESM.docx]

**Additional File 1.** Limit of detection for *eqbE* LAMP assay was determined via a “standard curve” as 0.005 ng of DNA using *S. equi* ATCC 33398.

| *S. equi* ATCC 33398 | *eqbE* LAMP | *eqbE* LAMP |
| --- | --- | --- |
| 500 ng | Pos | N/A |
| 50 ng | Pos | Pos |
| 5 | Pos | Pos |
| 0.5 ng | Pos | Pos |
| 0.05 ng | Pos | Pos |
| 0.005 ng | Pos | Pos |
| 0.0005 ng | Neg | N/A |
| 0.00005 ng | Neg | N/A |
| 0.000005 ng | Neg | N/A |
| 0.0000005 ng | Neg | N/A |
